# Minimizing the number of origins in batches of weaned calves to reduce their risks of developing bovine respiratory diseases

**DOI:** 10.1186/s13567-020-00872-z

**Published:** 2021-01-07

**Authors:** Thibaut Morel-Journel, Sébastien Assié, Elisabeta Vergu, Jean-Baptiste Mercier, Florence Bonnet-Beaugrand, Pauline Ezanno

**Affiliations:** 1 INRAE, Oniris, BIOEPAR, 44300 Nantes, France; 2 INRAE, Université Paris-SaclayMaIAGE, 78350 Jouy-en-Josas, France; 3 Terrena Innovation, La Noëlle, 44155 Ancenis, France

## Abstract

Bovine respiratory diseases (BRD) are a major concern for the beef cattle industry, as beef calves overwhelmingly develop BRD symptoms during the first weeks after their arrival at fattening units. These cases occur after weaned calves from various cow-calf producers are grouped into batches to be sold to fatteners. Cross-contaminations between calves from different origins (potentially carrying different pathogens), together with increased stress because of the process of batch creation, can increase their risks of developing BRD symptoms. This study investigated whether reducing the number of different origins per batch is a strategy to reduce the risk of BRD cases. We developed an algorithm aimed at creating batches with as few origins as possible, while respecting constraints on the number and breed of the calves. We tested this algorithm on a dataset of 137,726 weaned calves grouped into 9701 batches by a French organization. We also computed an index assessing the risks of developing BRD because of the batch composition by considering four pathogens involved in the BRD system. While increasing the heterogeneity of batches in calf bodyweight, which is not expected to strongly impact the performance, our algorithm successfully decreased the average number of origins in the same batch and their risk index. Both this algorithm and the risk index can be used as part of decision tool to assess and possibly minimize BRD risk at batch creation, but they are generic enough to assess health risk for other production animals, and optimize the homogeneity of selected characteristics.

## Introduction

Respiratory diseases are a major sanitary and economic concern for multiple farm animal industries, including pigs [1, 2], dairy [3, 4] and beef cattle [5, 6]. Bovine respiratory diseases (BRD) are also known as “shipping fever” in the young bull industry, because of their high incidence amongst young bulls in the early weeks after their transport to the fattening units (around 18.5% during their first 6 weeks in pens in France) [7, 8]. The economic impact of BRD has been estimated at $13,895 per 1000 animals in the United States [9], and around 20% of farmers’ revenues in France [10, 11]. The clinical signs of BRD are underpinned by complexes of pathogens [12, 13], including bacteria (e.g. *Mannheimia haemolytica*, *Pasteurella multocida*, *Histophilus somni*, *Mycoplasma bovis*) and viruses (e.g. the bovine respiratory syncytial virus, the bovine herpes virus-1 or the parainfluenza type 3 virus). The diversity of the involved pathogens hinders prevention measures and leads to a strong preventive use of antibiotics [14]. Besides prophylactic measures, the management of weaned beef calves up to fattening is another strategy that could be used to reduce their risks of developing BRD.

The French young bull industry involves two types of stakeholders [15]. On the one hand, cow-calf producers rear suckling beef calves with their dam until the age of five to ten months. On the other hand, fatteners purchase weaned calves from cow-calf producers and handle their fattening, before they are slaughtered, typically when they are 17 months old [15]. In fattening units, young bulls are managed by batches, i.e. groups of animals fattened together (feedlots). Instead of purchasing weaned calves separately, fatteners often use third-party intermediates, namely independent middlemen and producer organizations, who design the batches and sell them to the fatteners at once [16]. In order to manage large numbers of weaned calves, intermediates can use sorting centres, where the calves are sent, and assigned to batches. Besides the breed of the animals, homogeneity of animal bodyweight is the main criterion used to create batches because it is expected to facilitate their management and improve their performance, although this assumption has been questioned [17, 18]. Weaned calves are especially prone to developing BRD in the first weeks of fattening because the creation of batches mixes calves from various origins, i.e. cow-calf producers [18, 19]. Gathering calves from different origins not only generates additional stress, by disrupting existing hierarchies between individuals, but it also increases cross-contamination risks between calves potentially carrying different pathogens, some of them involved in the BRD complex. Limiting the mixing of individuals from different origins could therefore reduce the BRD incidence among weaned calves.

In this study, we investigated whether homogeneity in the origins of animals (i.e. the cow-calf producer they come from) could be a useful criterion to design batches, in order to reduce the risk of BRD occurrence among young bulls in the first weeks after their arrival in fattening units. First, we developed an algorithm indicating batch compositions minimizing the average number of different origins within each batch, while respecting additional constraints relative to the number, breed and date of arrival at the sorting centre of the weaned calves assigned to each batch. Second, we tested the algorithm on a dataset of batches created by a French beef producer’s organization. We compared the batches from the original dataset to the batches created by the algorithm in order to identify a decrease in the number of origins per batch and check the impact of the algorithm on other characteristics of the batches, namely the heterogeneity in the age or weight of the calves. Third, we computed an index for each calf of the dataset, representing its risk of developing symptoms because of four theoretical pathogens representing four of the main pathogens involved in BRD. We compared the risk index of the calves according to the batch composition given by the dataset to those according to the batch composition provided by the algorithm, in order to assess its impact on the estimated risk of developing BRD.

## Material and methods

### Presentation of the database

We used a dataset provided by *Terrena Production Bovine*, a beef producer organization located in Western France, a region densely populated with cattle. The data includes 137,726 weaned calves managed by the organization between 2010 and 2018, with information about their breed, age and weight. Most calves were Charolais bulls (60%), although a large diversity of other breeds—23 in total—was represented. In addition, the origin of each animal (i.e. the cow-calf producer it comes from), the batch it was assigned to and the date of the assignment were also provided. All the calves in the database were managed by the same intermediate (*Terrena Production Bovine*). They came from 3675 different cow-calf producers and were sold to 1028 different fatteners. The cow-calf producers were very unevenly represented in the database: the average cow-calf producer provided 37.3 calves, but 52% of them provided 10 calves or less while 5.2% of them provided 100 calves or more. Overall, 10% of the cow-calf producers provided 66.5% of the calves. A very large majority (94.8%) of the calves were not sent directly from the cow-calf producer to the fattener, but went through at least one sorting centre belonging to *Terrena Production Bovine*. The average duration of the calves' stay in the sorting centres was 2.44 day (standard deviation of 2.78 days), with 33% of the calves staying 1 day (i.e. they arrived and exited the centre on the same day) and 97% of them staying 4 days or less. This is consistent with the management of the organization, which is organized on the first 4 days of the weeks: most calves (62%) arrive in centres on Mondays or Tuesdays and most (80%) exit on Wednesdays or Thursdays.

From this database, we extracted the following information about the 9,701 batches these calves were assigned to: their size (i.e. the number of animals in each batch), their date of creation, the main breed represented in each batch. The batches were defined according to the database and corresponded to groups of all calves sent to a same fattener on the same day. The average batch included 14.15 calves (standard deviation of 11.9 calves) and 50% of the batches regrouped between 6 and 19 calves. We considered four classes of proportion of calves of the main breed in each batch, which are those used by *Terrena Production Bovine*: [0;0.5], [0.5;0.9], [0.9;1] and 1 (all the animals were of the main breed). Batches in the class [0;0.5] were considered to have no main breed. Finally, we computed three additional characteristics for each batch: the number of different origins represented, the standard deviation in age and the standard deviation in weight of the animals.

### Presentation of the optimization algorithm

**Figure 1:**
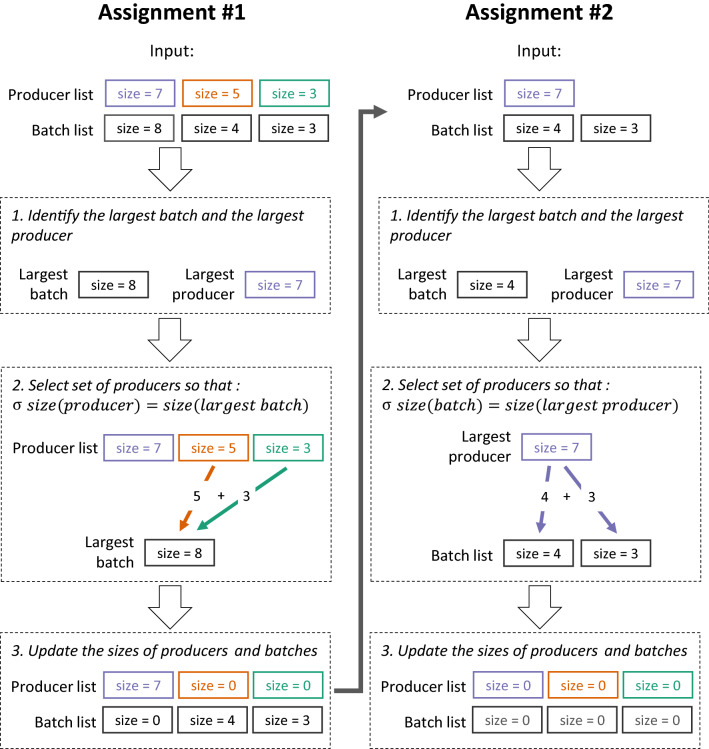
**Schematic representation of two successive assignments performed by the algorithm.** During the assignment #1, the largest batch is larger than the largest producer, so the algorithm selects a set of two producers (of sizes 5 and 3). During the assignment #2, the largest producer is larger than the largest batch, so the algorithm selects a set of two batches (of sizes 4 and 3). The updated producer and batch lists at the end of the assignment #1 is used as the input of the assignment #2.

The management of beef calf batches by intermediates involves two sets of elements. On the one hand, there is a set of cow-calf producers, each characterized by the number of calves they can provide (its “size”, noted *X*_*a*_ for cow-calf producer *a*). On the other hand, there is a set of batches, each characterized by the number of calves they can receive (its “size”, noted *Y*_*b*_ for batch *b*). The sizes *X*_*a*_ and *Y*_*b*_ are fixed at values given in the database from *Terrena Production Bovine*. In this context, the objective of the algorithm is to assign calves to a single batch each, while minimizing the number of different cow-calf producers providing calves to a same batch. This functioning is akin to a wiring algorithm [20], although on a weighted bipartite graph. Indeed, the system described can be represented as two sets of vertices (the cow-calf producers and the batches). In this context, a cow-calf producer shares an edge with a batch only if at least one calf provided by the former is assigned to the latter. Then, the weight of the edge corresponds to the number of calves assigned. The wiring (i.e. edge structure of the graph) from the algorithm therefore corresponds to the composition in calves of all the batches. It is created by iterating the following procedure (Figure 1):

1. The algorithm orders the two sets in decreasing order of sizes (*X*_*1*_ and *Y*_*1*_ then correspond respectively to the size of the largest cow-calf producer and the size of the largest batch).

2a. If $X_{1} > Y_{1}$, the algorithm defines a objective value *v*, with $v = X_{1}$ initially. Then, it searches downward in the set of batches for the smallest subset of batches whose sum of sizes equals *w.* If multiple subsets are possible, the one selected is the one including the largest value of *Y*_*b*_. If there is no such subset, the algorithm decreases the value of *v* by one and tries again. This ensures that $v \le X_{1}$, i.e. that the largest cow-calf producer will always provide enough calves to create all the batches of the subset. When a subset is found, *v* calves from the largest cow-calf producer are assigned to the batches of the subset, the sizes of the batches are set to 0 and the size of the largest cow-calf producer is set to $X_{1} - v$.

2b. If $X_{1} \le Y_{1}$, the algorithm defines a objective value *v*, with $v = Y_{1}$ initially. Then, it searches downward in the set of cow-calf producers for the smallest subset of cow-calf producers whose sum of sizes equals *v*. If multiple subsets are possible, the one selected is the one including the largest value of *X*_*a*_. If there is no such subset, the algorithm increases the value of *v* by 1 and tries again. This ensures that $v \ge Y_{1}$, i.e. that the cow-calf producers of the subset will always provide enough calves to create the largest batch. When a subset is found, the smallest cow-calf producer of the subset is noted *z* and its size *X*_*z*_. All the calves from all cow-calf producers of the subset but *z* are assigned to the largest batch, as well as $X_{z} - \left( {v - Y_{1} } \right)$ calves from *z*. Then, the size of the largest batch and all cow-calf producers of the subset but *z* are set to 0 and *X*_*z*_ is set to $v - Y_{1}$.

If no other constraint is set, the algorithm continues until the sizes of either all cow-calf producer or all batches are equal to 0.

For this study, we included two other constraints in order to recreate the batches of the database from *Terrena Production Bovine*: (i) assigning calves provided a given week to batches created the same week, and (ii) respecting the main breed and class of proportion of the batches generated. To respect the first constraint, the algorithm performed the assignments chronologically on a weekly basis. For each week, the sizes of each cow-calf producer and of each batch were set as the number of calves the provided or received on the given week. If there was an excess or deficit of calves provided, an option in the algorithm made it possible to carry the remaining cow-calf producers or the remaining batches over to the next week. Their sizes at the end of a weekly assignment were then simply added to their sizes at the next week. The impact of the size of the time-window was investigated in Additional file 1.

**Figure 2:**
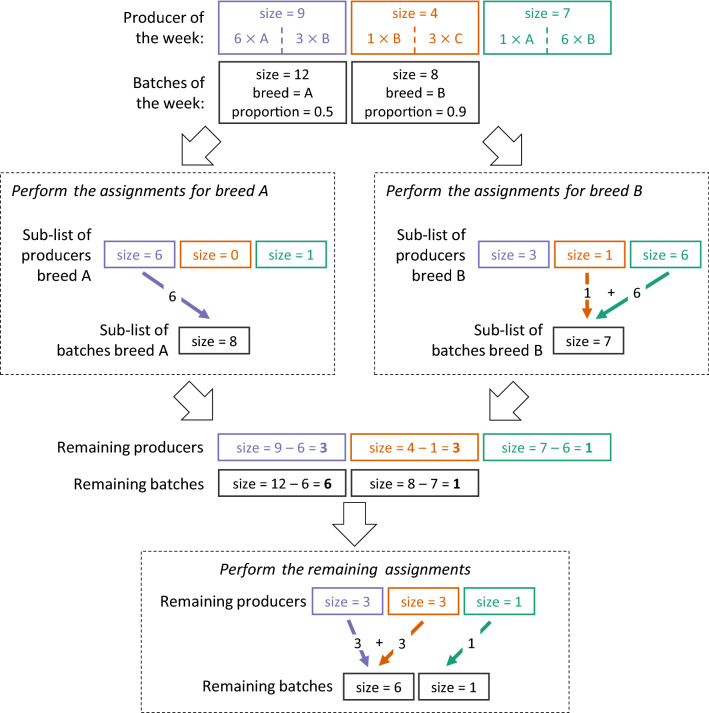
**Schematic representation of sub-lists management by the algorithm to respect the constrains.** The algorithm starts by ensuring that enough calves of the given breed are assigned to the batches, by assigning the 6 required calves of breed A and the 7 required calves of breed B. Then, it computes the list of remaining calves at the producers and the remaining required calves for the batches, and perform the remaining assignments.

To respect the second constraint, the algorithm first performed assignments separately for each breed identified as the main breed of at least one batch in the set considered (Figure 2). Before each of these assignments, the sizes of all the cow-calf producer were set to the number of calves they provided from the specific breed, the sizes of the batches with this main breed were set at the minimal number of calves required to match their class of proportion, and the sizes of all the batches with another main breed than the one considered were set to 0. After, the algorithm performed the remaining assignments regardless of breed with the complete sets of batches and of cow-calf producers, whose sizes were updated to account for the calves already assigned.

### Computation of the risk of developing bovine respiratory symptoms

Our estimation of the risk that calves developed BRD symptoms during the first week of fattening *R*_*i,s,b*_ depended on (i) the batch composition (noted *c*), i.e. the way the calves were assigned to the batches, and on (ii) the sanitary situation (noted *s*), which was associated with a matrix indicating the presence of each pathogen at each cow-calf producer. Each element of the matrix, noted *q*_*j,k*_ was equal to 1 if pathogen *k* was present at cow-calf producer *j*, and 0 otherwise. From this matrix, we defined *Q*_*i,s,k*_ as a variable indicating whether calf *i* had encountered pathogen *k* at its cow-calf producer, and was therefore potentially carrying this pathogen. In the absence of individual-level information about the actual status of each calf (i.e. whether it was carrying the pathogen or not), the potential of each calf is considered identical and $Q_{i,s,k} = q_{j,k}$ if calf *i* originated from cow-calf producer *j*. We also defined *E*_*i,s,c,k*_ as the probability that calf *i* was exposed to pathogen *k* in its batch:

$$E_{i,s,c,k} = 1 - e^{{ - \alpha_{k} U_{i,s,c,k} }}$$

with α_*k*_ the parameter describing the specific risk of exposure associated with pathogen *k* and *U*_*i,s,c,k*_ the proportion of calves other than calf *i* in its batch which have encountered pathogen *k* at their cow-calf producer, computed as follows:

$$U_{i,s,c,k} = \mathop \sum \limits_{g = 1}^{{N_{i,c} - 1}} \frac{{Q_{g,s,k} }}{{N_{i,c} - 1}},g \ne i$$

with *N*_*i,c*_ the size of the batch of a calf *i* and way of creating batches *b*. The value of *E*_*i,s,c,k*_ was always lower than 1, and was equal to 0 if $U_{i,s,c,k} = 0$, i.e. if no calf in the batch had encountered pathogen *k*. The value of *R*_*i,s,c*_ was computed as follows:

$$R_{i,s,c} = \mathop \sum \limits_{k} \mathop \sum \limits_{w} r_{i,s,c,k,w}$$

with *r*_*i,s,c,k,w*_ the specific risk that calf *i* developed symptoms because of pathway *w* of pathogen *k*. We considered three different pathways leading to the development of BRD. The first one was the naïve infection ($w = na\"i ve$), i.e. when the calf developed symptoms because of a pathogen not encountered at its cow-calf producer. The specific risk was computed as follows:

$$r_{i,s,c,k,na\"i ve} = \beta_{k} E_{i,s,c,k} \left( {1 - Q_{i,s,k} } \right)$$

with β_*k*_ the naïve infection parameter. The second way was the reinfection ($w = reinf$), i.e. when the calf developed symptoms because of a pathogen already encountered at its cow-calf producer. The specific risk was computed as follows:

$$r_{i,s,c,k,reinf} = \gamma_{k} E_{i,s,c,k} Q_{i,s,k}$$

with γ_*k*_ the reinfection parameter. The third way was the reactivation ($w = react$), i.e. when the calf developed symptoms because of a pathogen previously encountered, regardless of the way of creating batches. The specific risk was computed as follows:

$$r_{i,s,c,k,react} = \delta_{k} Q_{i,s,k}$$

with *δ*_*k*_ the reactivation parameter.

We considered a complex of four theoretical pathogens, each representing one of main pathogens involved in the BRD complex: *M. haemolytica* (Mh) and *M. bovis* (Mb) the bovine respiratory syncytial virus (BRSV) and the bovine parainfluenza-3 virus (PI-3). The values of *α*_*k*_, *β*_*k*_, *γ*_*k*_ and *δ*_*k*_ for each pathogen are listed in Table 1. For the sake of clarity, their values were all integers. They only mattered relative to one another, but were chosen to reflect the general behaviour of their real counterparts [7, 21, 22]. For instance, the values of *α*_*Mh*_ and *β*_*Mh*_ were respectively chosen to represent the low risks associated with being exposed to *M. haemolytica* and its high virulence for naïve calves. Similarity, the value of *α*_*Mb*_ reflected the much higher risk of being transmitted of *M. bovis* and the values of *δ*_*BRSV*_ and *δ*_*PI-3*_ were null to reflect the absence of reactivation risks of the two viruses. The actual sanitary situation of the farms listed in the *Terrena Production Bovine* database was unknown. Therefore, we generated two sets of 10,000 sanitary situations (*s*) according to two scenarios of pathogen distribution across the farms. The first scenario assumed no correlation in the presence of pathogens at the cow-calf producers: each value of *q*_*j,k*_ was randomly drawn according to a Bernoulli distribution of parameter *π*_*k*_ (Table 1). The second scenario assumed a positive correlation in the presence of the pathogens: each value of *q*_*j,k*_ was randomly drawn according to a Bernoulli distribution whose parameter depended on the number of other pathogens present (see Additional file 2). In both scenarios, the average probability of presence of each pathogen across all farms was equal to its seroprevalence, as defined from the literature [7, 22].

**Table 1:** Values of seroprevalence (*π*_*k*_), exposure parameter (*α*_*k*_), naïve infection parameter (*β*_*k*_), reinfection parameter (*γ*_*k*_) and reactivation parameter (*δ*_*k*_) of the four theoretical pathogens considered for this study (Mh, Mb, BRSV and PI-3)

	Mh	Mb	BRSV	PI-3
*π*_*k*_ (seroprevalence)	0.79	0.46	0.32	0.62
*α*_*k*_ (exposure)	5	20	10	10
*β*_*k*_ (naïve infection)	10	8	10	7
*γ*_*k*_ (reinfection)	2	4	4	3
*δ*_*k*_ (reactivation)	3	6	0	0

The average risk over all sanitary situations was noted *R*_*i,∙,c*_. We considered two batch compositions: the historical one ($c = hist$) corresponded to the assignment of calves to batches as described in the *Terrena Production Bovine* database, while the optimized one ($c = opti$) corresponded to the assignments performed by our algorithm. The difference between the risk indices for the two batch compositions was noted $D_{i,s} = R_{i,s,opti} - R_{i,s,hist}$ and its average over all sanitary situations was noted *D*_*i,∙*_. Finally, we defined the threshold risk index *h*_*s,p*_ over which calves were expected to develop BRD, for a sanitary situation *s* and an observed proportion of calves developing symptoms *p*. The value of *h*_*s,p*_ corresponded to the (1-*p*)th percentile of *R*_*i,s,hist*_. From *h*_*s,p*_, we could derive the proportion of calves expected to develop BRD with the optimized batch composition *p *^‘^_*s,p*_ for the same sanitary situation and observed proportion of calves developing symptoms. Then, the relative change in proportion of calves expected to develop symptoms after optimization was $\frac{{p_{s,p}^{^{\prime}} - p}}{p}.$

## Results

### Characteristics of the batches

**Figure 3:**
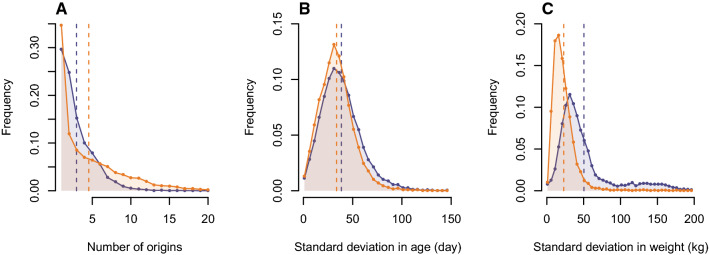
**Batch characteristics for the historical and optimized way of creating batches.** Distribution of the number of origins (**A**), the standard deviation in age (**B**) and the standard deviation in weight (**C**) per batch, for the historical (orange) and optimized data (purple), with the mean value of the two distributions (vertical dotted line).

The comparison between the *Terrena Production Bovine* assignments and the optimized ones showed a decrease in the number of origins per batch (Figure 3A), from 4.55 to 2.95 origins on average. Especially, we observed much fewer batches with a very large number of different origins. Indeed, the proportion of batches with more than 5 and 10 origins were reduced from 31.4 to 12.0% and from 10.8 to 0.8% respectively. Conversely, we observed a trade-off on the lowest number of origins: there were fewer batches with a single origin and more with between two and four origins after optimization. The other criteria usually considered for the creation of batches were also affected by the optimization. On the one hand, the standard deviation in age of the batches increased slightly from 33.7 to 38.2 days on average (Figure 3B), although the peak of the distribution remained around a 30-day standard deviation. On the other hand, the standard deviation in weight increased from 23 to 50 kg on average (Figure 3C). In addition, the peak of the distribution shifted towards greater standard deviations, and the distribution displays a thicker tail of batches with a high standard deviation.

### Risk index

**Figure 4:**
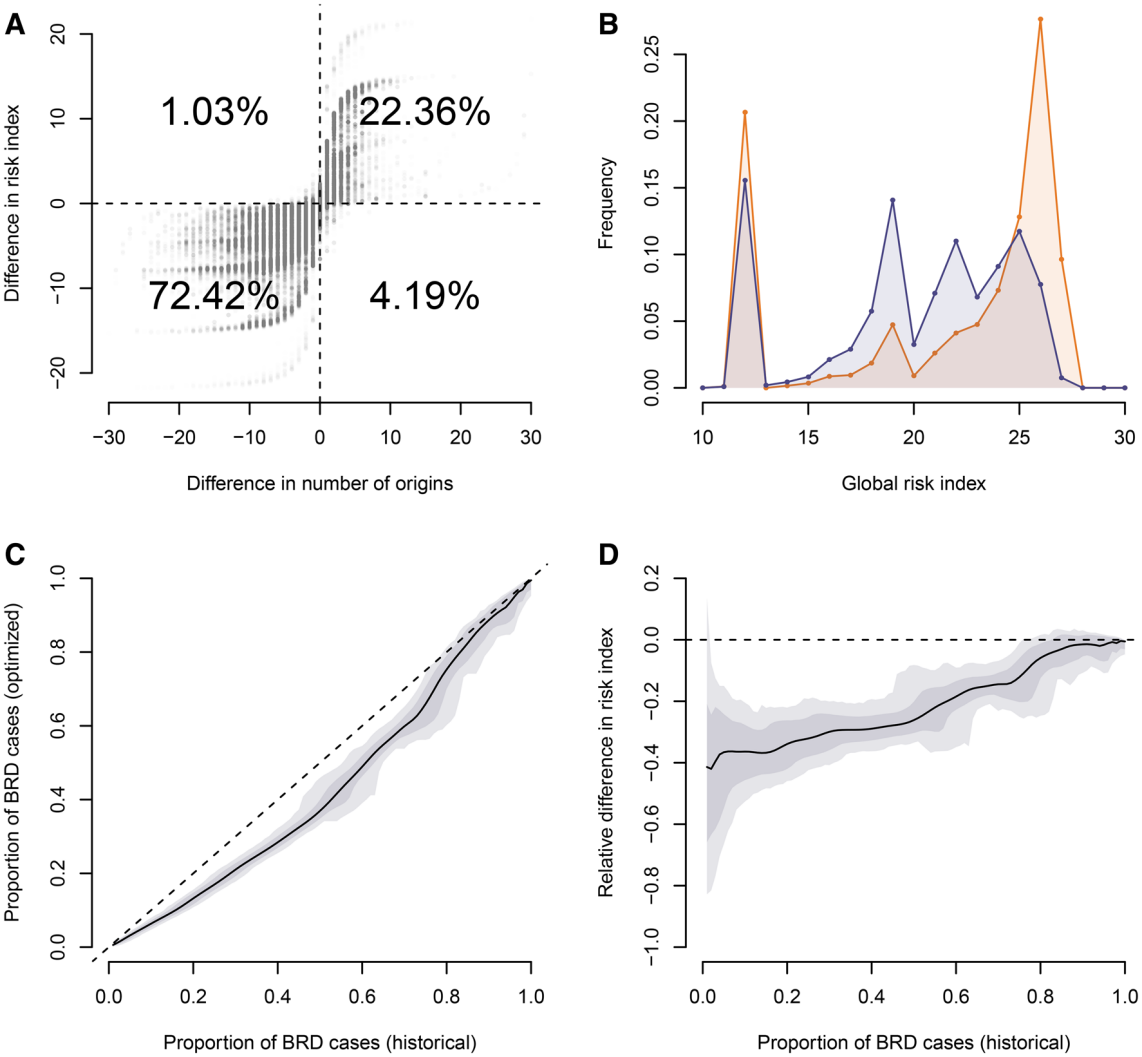
**Results concerning the risk indices for the historical and optimized way of creating batches.** **A** Difference between the risk indices with the optimized batch composition and those with the historical batch composition, as a function of the difference in number of origins, with the proportion of data points in each quadrant. **B** Distribution of the risk indices for the historical (orange) and optimized batch compositions (purple), with the mean value of the two distributions (vertical dotted line). **C**, **D** Average proportion of calves expected to develop BRD $p^{\prime}_{s,p}$ (**C**) and the relative change in the number of calves developing symptoms (**D**) as a function of the observed proportion of calves currently developing symptoms $p$ (solid line), with envelops including 50% (dark grey) and 90% (light gray) of the 10,000 sanitary situations.

There was almost no difference in the values of *R*_*i,∙,hist*_ and *R*_*i,∙,opti*_ (the risk indices averaged over all sanitary situations, respectively for historical and optimized batch compositions) for the two scenarios with and without correlation in the presence of pathogens at the cow-calf producers (see Additional file 2). The results presented here focus on the scenario assuming no correlation. We found that the change in the number of origins was strongly correlated with the average change in the risk index of calves after optimization *D*_*i,∙*_ (Spearman’s *ρ* = 0.85, p < 0.0001). Hence, changes in the risk index were positively correlated with changes in the number of origins for 94.8% of the calves (Figure 4A). The distributions of *R*_*i,∙,hist*_ and *R*_*i,∙,opti*_ (the risk indices averaged over all sanitary situations, respectively for historical and optimized batch compositions) were comprised between 11 and 28, with respective means at 21.53 and 20.07 (Figure 4B). The distribution of *R*_*i,∙,hist*_ exhibited two peaks, respectively at low (12) and at high risk values (26). The distribution of *R*_*i,∙,opti*_ only exhibited a low-risk peak, albeit lower, while the rest of the values was more evenly distributed. The value of *p *^‘^_*∙,p*_ (the proportion of calves expected to develop BRD with the optimized batch composition averaged over all sanitary situations) were systematically lower than *p* (the observed proportion of calves developing BRD) for any value of *p* between 0.01 and 1 (Figure 4C). The relative change in proportion of calves expected to develop symptoms after optimization was also strong, especially for lower values of *p*, with an average decrease of more than 20% if $p \le 0.57$ and more than 30% if $p \le 0.29$.

To better understand how the algorithm impacted the risk index depending on the characteristics of the batches, we divided our set of 9701 batches into classes of (i) size, (ii) proportion of calves of the main breed, (iii) number of origins according to the database and (iv) weight homogeneity according to the database, and computed at the average values of *R*_*i,∙,hist*_ and *R*_*i,∙,opti*_ for each class (see Additional file 3). Results show that the algorithm first and foremost reduced the average risk index of calves in the largest batches and those with the highest proportion of calves of the same breed. While the algorithm also decreased the average risk in batches with originally many origins and high weight heterogeneity, it actually increased the average risk of the batches with the smallest number of origins and the most homogeneous weight originally.

**Figure 5:**
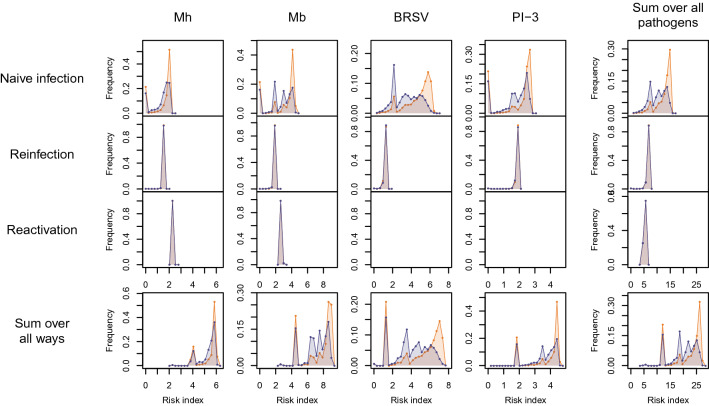
**Specific risks associated with each way of infection for each pathogen.** Distribution of the specific risks associated with each pathogen (by column) and each way of infection (by row) for the historical (orange) and optimized batch compositions (purple), with partial sums over all pathogens (leftmost column) and over all ways (bottom column) and the risk index distribution (bottom right).

To assess the impact of the parameter values defining the characteristics of the pathogens on the relationship observed between *R*_*i,∙,hist*_ and *R*_*i,∙,opti*_, we also computed the risk indices after changing values of either *α*_*k*_, *β*_*k*_, *γ*_*k*_ or *δ*_*k*_ for either pathogen (see Additional file 4). Results show that these variations did have an impact on the absolute values of the indices, but that the general shape of the distributions remained similar. Especially, the values of *R*_*i,∙,opti*_ always remained lower than those of *R*_*i,∙,hist*_. Besides, we also assessed the respective contributions of each specific risk associated with each way of infection and each pathogen on the risk index. The distributions of *r*_*i,∙,hist,k,naïve*_ and *r*_*i,∙,opti,k,naïve*_ (the specific risks of naïve infection averaged over all sanitary situations, respectively for the historical and optimized batch composition) strongly differed for every pathogen, but this was not the case for the specific risks associated with reinfection and with reactivation (Figure 5). Hence, almost all the differences between the risk indices with the historical and optimized batch compositions came from differences in the naïve infection risks.

## Discussion

In this study, we showed that batches could be systematically reorganized to reduce the overall number of different origins of the calves in each batch, while respecting multiple constraints on the number and breed of the calves. In addition to reducing the average number of origins by 35%, the algorithm limited the maximum number of origins in the batches, with few batches created with calves originating from more than five different cow-calf producers. The reduction in the number of origins was highly correlated with the risk index, and expected to result in fewer BRD cases during fattening. In particular, the algorithm reduced the specific risks of naïve infection, i.e. the risks of exposure of weaned calves to pathogen with which they had never been in contact before the creation of the batch. Assuming that the observed proportion of BRD cases in the *Terrena Production Bovine* dataset corresponds to the incidence of 18.5% estimated in a previous study concerning France [7] and that our risk index accurately represents the risks of developing BRD, we expect 12.4% of cases with the optimized batch. Such a reduction of 35% would not only improve the health of cattle, but also their performance during fattening, as it is also impacted by BRD [9].

Analysing more specifically how the algorithm affected the risk index depending on batch characteristics revealed two mains impacts (see Additional file 3). First, the algorithm preferentially improved the largest batches and the batches with the most calves of the main breed, which is consistent with the priority order in the creation of batches. Second, it has also improved more batches with the greatest heterogeneity in weight and the most different origins according to the database from *Terrena Bovine Production*, i.e. those with a priori the greatest potential for improvement. However, the average risk index increased for the batches with the lowest number of origins (one or two) in the original database. This trade-off is consistent with the observed decrease in the number of batches with one or two origins, although the average risk index of these batches remained lower than those of the other classes of batch size. Besides, the improvement brought about by the algorithm remained visible for batches with as few as three different origins. This trade-off was also expected, and is at the core of a more general compromise between the goal of the cooperatives—globally decreasing the risks of developing BRD across all batches—and the individual goal of the fatteners, who are already minimizing the BRD risks in their own fattening units. Defining which goal should outweigh the other is a question beyond the scope of this study, but related to the more general changes in practices induced by the use of such algorithms.

The risk index we designed for this study allowed us to assess how mixing calves from different origins affects their risks of developing symptoms. Information is still lacking on the impact of the different pathogens included in the BRD complex and their interactions. We therefore used a simplified complex of four theoretical pathogens without any interactions taken into account in the risk index. As the risk index was an algebraic sum of the specific risks of each pathway for each pathogen, we were able to identify precisely the origin of the difference between the risk indices from the historical and the optimized batch compositions by considering the values of risks specific to a pathway or a pathogen. The results mainly showed differences in the probability of naïve infection, which were consistent with the algorithm’s reduction of origin mixing. Indeed, by encouraging interactions between calves coming from the same cow-calf producers, the risks of cross-contaminations by pathogens never encountered was directly limited. The reduced risks of naïve infection were not compensated by higher risks of reinfection, although reinfection by an already encountered pathogen was more likely when multiple calves of the same origin were in the same batch. This can be explained by the high probability that calves were usually already in a batch with others from the same origin, even with the historical batch composition. Interestingly, the positive correlation in the presence of pathogens at cow-calf producers seemed to have almost no impact on the value of the risk index (see Additional file 2). This is likely because the impact of the correlation was averaged over the 10,000 sanitary situations generated. Even though the scenario with positive correlation in the presence of pathogens included more cow-calf producer with either 0 or 4 pathogens, each cow-calf producer was equally likely to be part of the producers with no pathogen or part of the producers with all the pathogens. Empirical data on the biosecurity level of each cow-calf producer would be needed to preferentially attribute the pathogens to specific cow-calf producers, and potentially show an impact of the correlation in pathogen presence.

Empirical data was also too scarce for now to confirm the observed impact of our algorithm, or to develop a more comprehensive model describing precisely the modes of action and interactions of the pathogens of the bovine respiratory disease complex. Thus, we deliberately chose to present a risk index that could be easily used to estimate potential risks associated to the batch composition. Yet, it appears that the impact of our algorithm on risks of developing BRD is rather robust to changes in the values of the four parameters of exposure (*α*_*k*_), naïve infection (*β*_*k*_), reinfection (*γ*_*k*_) and reactivation (*δ*_*k*_) (see Additional file 4). Especially, the risk index with the optimized batch composition remained systematically lower than the one with the historical batch composition. Therefore, this effect is unlikely to be an artefact of the specific parameter values considered for the pathogens in the study. Moreover, our results were also largely independent of the original proportion of calves developing BRD during the first week of fattening. Indeed, we observed a relative decrease of more than 20% in the proportion of calves expected to develop BRD with the optimized batch composition, for all values of *p* (the observed proportion of calves developing BRD) between 1 and 57%. Thus, potential errors in the estimation of this observed proportion should have little impact on our conclusions regarding the efficiency of the algorithm. This also indicates that we should expect similar effects of the algorithm in other contexts, where the initial proportion of animals developing symptoms is different.

The use of such algorithms brings about potential evolutions in the way intermediates design batches. First, farmers, middlemen and organizations may have to revaluate the relevance of weight homogeneity as a criterion for batch creation. Indeed, our algorithm not only significantly increased the standard deviation of the batch weights, but also created a small proportion of batches with particularly high weight heterogeneity. This trade-off was not surprising, as weight homogeneity was the main criterion used for the historical batch composition by *Terrena Production Bovine*, whereas it was not taken into account at all for the optimized batch composition from the algorithm. Even though weight homogeneity is not expected to have a strong impact on growth performance during fattening [17–19], abandoning this criterion would nevertheless have to be accepted by the different stakeholders, especially fatteners. Second, it may encourage intermediates to get information about available calves to create batches in advance. If information from fatteners on the required batches is easily accessible, knowing in advance which calves will be available can be more difficult. Indeed, multiple intermediates are potentially competing for calves from cow-calf producers, leading to uncertainty about which calves they will actually manage. This lack of information could prevent users of the algorithm from expanding the time-window, which would, however, improve the performance of the algorithm. We ran the algorithm with a time-window from 1 day to 6 weeks, in order to assess the impact of the time-window size on the efficiency of the algorithm (see Additional file 1). The mean number of origins per batch appeared to tend to an asymptotic value between 2 and 2.5 origins as the size of the time-window increased. The 7-day window we used has already led to a sizeable reduction to 2.95 origins on average, while still manageable for a producers’ organizations. The marginal improvement expected by further increasing the size of the window may therefore not be sufficient compared to the associated logistical costs of getting information from cow-calf producers further in advance.

In this study, we tested our algorithm retrospectively on an existing database, and used the risk index we present to assess the expected impact of our algorithm on the probability of weaned calves developing BRD. However, these two tools are readily available, and could also be used by cooperatives and other intermediaries on a daily basis. On the one hand, the algorithm can be used as is, as part of decision support tools to design batches of young calves with less mixing of origins. On the other hand, the risk index can help quantifying the risks associated with given batch compositions, and possibly identify the batches most likely to present cases of BRD before the actual batches are even created. These decision-making tools should be used in addition to other practices aimed at limiting the need for prophylactic measures during fattening by limiting BRD risks. These practices include promoting the pre-weaning vaccination of calves and minimizing transportation distances. Vaccination prevents the development of symptoms in two ways: by improving the resistance of calves to BRD and by reducing shedding [23]. Yet, it is most efficient when it is carried out early in the animal’s life, in order to enable the development of immunity prior to first weeks of fattening [24, 25]. This places the economic burden of vaccination on cow-calf producers without much incentive for them to do so, as it mainly benefits fatteners. In this context, intermediates could promote vaccination (and other preconditioning practices) by generally informing about the vaccination status of the animals and participating in the implementation of premiums for vaccinated calves (for buying from producers and for selling batches), which are still scarce in the beef industry [26, 27]. The impact of the distance travelled on the calves’ health is well documented [28–30]: transportation is a stressful event for the weaned calves, which increases their risks of developing respiratory diseases once they arrive in fattening units. Therefore, minimizing the distance travelled by the animals is a complementary way for intermediates to act on BRD risks. Optimizing the itineraries in order to minimize transportation distances appears as the next logical step after the optimization of the batch composition, and could even be improved by the latter. Indeed, our algorithm could facilitate direct transfers from cow-calf producers to the fattening units, by creating batches with calves coming from few or a single cow-calf producer, without the need to sending them to sorting centres.

## Supplementary Information

**Additional file 1.** Efficiency of the algorithm as a function of time-window size.

**Additional file 2.** Impact of the positive correlation between pathogen presence at the cow-calf producer.

**Additional file 3.** Impact of the algorithm on the risk index depending on batch characteristics.

**Additional file 4.** Robustness of the results to changes in parameter values.

## Data Availability

The code of the algorithm, as well as the script used to compute the global risk index and all other measures used in the manuscript and a test dataset are freely available at: https://sourcesup.renater.fr/projects/pub-youngb-algo. The dataset analysed for the study are the propriety of *Terrena Production Bovine* and contain commercial data which are not publicly available.

## Abbreviations

BRD: bovine respiratory diseases
BRSV: bovine respiratory syncytial virus
Mh: *Mannheimia haemolytica*
Mb: *Mycoplasma bovis*
PI-3: bovine parainfluenza-3 virus
