# Supplementary material for: Minimizing the number of origins in batches of weaned calves to reduce their risks of developing bovine respiratory diseases
Source: Vet Res. 2021 Jan 7;52:5. doi: 10.1186/s13567-020-00872-z (PMC7792323; doi:10.1186/s13567-020-00872-z)
Supplement: Supplementary file 1 — Additional file 1. Efficiency of the algorithm as a function of time-window size. [file 13567_2020_872_MOESM1_ESM.pdf]

Additional file 1: Efficiency of the algorithm as a function of time-window size

We investigated how the size of the time-window considered by the algorithm impacted its efficiency, i.e. its ability to reduce the mean number of origins in each batch. To do so, we ran the algorithm in the exact same conditions as those presented in the main text of this article, but with another time-window size. We considered six time windows smaller than the size used in the main study – from one to six days – and five time windows greater than the one used – from two to six weeks. For each time-window size, we ran the algorithm with or without the possibility of carrying over calves and batches to the next time-window.

We computed the mean number of origins for each run and plotted them against the time-window size (Figure S1), with the dots as the mean number of origins and the dotted horizontal line as the mean number of origins according to the historical batch composition of the *Terrena Production Bovine* dataset.

The results show that the mean number of origins was always substantially lower than the historical value. For time-windows longer than 7 days, the mean number of origins seemed to tend towards a minimal asymptotic value of between 2 and 2.5 origins per batch. The results for time-windows less than 7 days depended on the possibility to carry over unassigned calves and batches to the next time-window. The mean number of origins remained similar with this possibility, but continued to increase as the time-window decreased without this possibility.

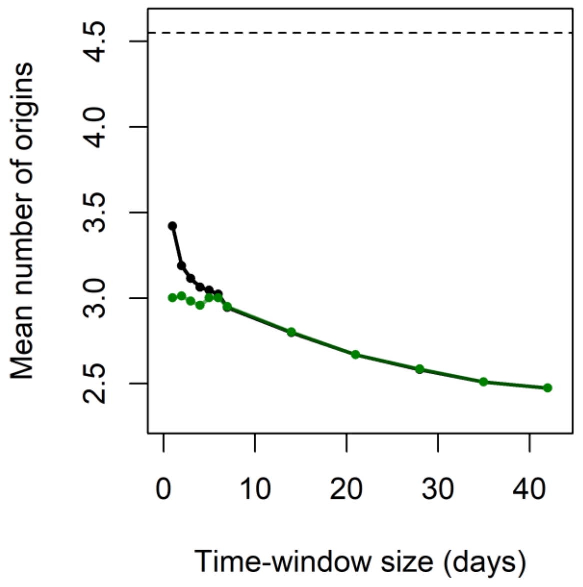

Figure S1: Mean number of origins of the batches after optimization by the algorithm depending on the time-window size used, with (green) or without (black) the possibility to carry over unassigned calves and batches to the next time-window. The dashed horizontal line corresponds to the mean number of batches from the database from *Terrena Production Bovine*.
