# Supplementary material for: Minimizing the number of origins in batches of weaned calves to reduce their risks of developing bovine respiratory diseases
Source: Vet Res. 2021 Jan 7;52:5. doi: 10.1186/s13567-020-00872-z (PMC7792323; doi:10.1186/s13567-020-00872-z)
Supplement: Supplementary file 2 — Additional file 2. Impact of the positive correlation between pathogen presence at the cow-calf producer. [file 13567_2020_872_MOESM2_ESM.pdf]

Additional file 2: Impact of the positive correlation between pathogen presence at the cow-calf producer

Generation of the sanitary situations

As explained in the main text of the article, a sanitary situation corresponds to a matrix whose elements $q_{j,k}$ describe the presence ($q_{j,k}$ = 1) or absence ($q_{j,k}$ = 0) of pathogen k at cow-calf producer j. For a sanitary situation s, the values of $q_{j,k}$ are randomly drawn according to a Bernoulli distribution, successively for each pathogen. The order in which the pathogens are considered is itself randomized for each sanitary situation.

In a scenario without correlation between the values of $q_{j,k}$ for a same cow-calf producer j, the parameter of the Bernoulli distribution is π_k, the seroprevalence of pathogen k (as indicated in Table 1 from the main text of the article).

In a scenario with a positive correlation between the values of $q_{j,k}$ for a same cow-calf producer j, the parameter $P_{j,k}$ of the Bernoulli distribution of cow-calf producer j had to : (i) increase linearly with $S_{j,k}$, the number of pathogens already present in this cow-calf producer, (ii) be comprised between 0 and 1 and (iii) be chosen so that the average of $P_{j,k}$ over all the cow-calf was equal to π_k. To do so, we computed two values as follows:

$$P_{1,j,k} = \pi_k + C_{j,k}\left(\frac{1-\pi_k}{\max(C_{j,k})}\right)$$

$$P_{2,j,k} = \pi_k - C_{j,k}\left(\frac{\pi_k}{\min(C_{j,k})}\right)$$

with $C_{j,k}$ the centred value of $S_{j,k}$ ($C_{j,k} = S_{j,k} - S_{.,k}$). By definition, both $P_{1,j,k}$ and $P_{2,j,k}$ respect the conditions (i) and (iii). However, either one of them is comprised between 0 and 1 for all cow-calf producers. The one that meets this conditions is the parameter used in the Bernoulli distribution.

Example of the difference between cases with and without correlation

The following is an example of random draw of the values of $q_{j,k}$ for the two cases: with or without a positive correlation between the presence of the pathogens at the cow-calf producers.

Let's assume a set of 288 cow-calf producers, with already between 0 and 3 pathogens (i.e 0 < $q_{j,k}$ < 3). The values of $S_{j,k}$ are distributed as shown in Figure S2. The pathogen for which we draw the presence or absence has a seroprevalence π_k = 0.7. For the scenario without correlation, the values of $q_{j,k}$ are drawn from a Bernoulli distribution with this probability π_k for every cow-calf producer. The result of 10,000 random draws of this fourth pathogen is shown in Figure S3 (light blue). On average, the new pathogen is present in a similar proportion of cow-calf producer in every class of $S_{j,k}$ (i.e. equal to 0, 1, 2, or 3).

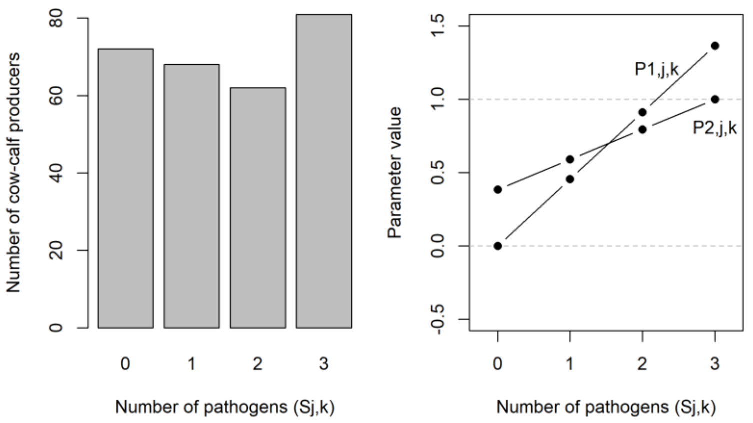

Figure S2: Number of producers in each class of $S_{j,k}$ values (left) and values of $P_{1,j,k}$ and $P_{2,j,k}$ (right) for our example

For the scenario with correlation, the values of $P_{1,j,k}$ exceed 1 for $S_{j,k}$ = 3, while the values of $P_{2,j,k}$ remain between 0 and 1. Therefore, the latter is selected as the parameter for the Bernoulli distribution. The results are also shown in Figure S3 (yellow) and show that the fourth pathogen is much more often present in at the cow-calf producers with a high value of $S_{j,k}$.

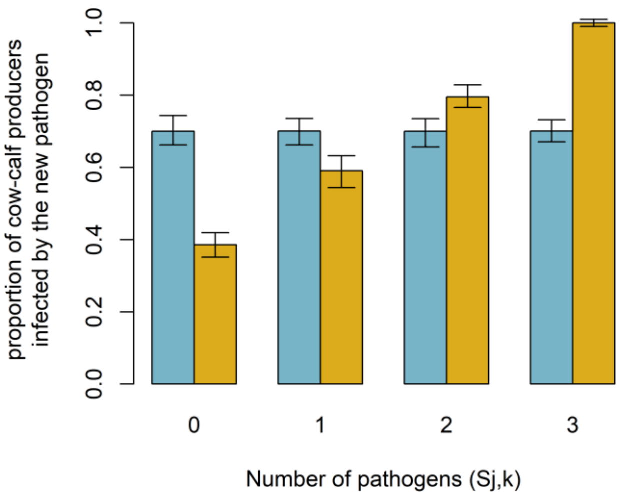

Figure S3: Average of 10,000 random draws for a case with (yellow) and without correlation (light blue). The bars represent the first and fourth quartile of the distribution of the random draws.

Differences in risk indices between the sanitary situations with or without correlation

The main text of the article presents a set of 10,000 sanitary situations generated without correlation to compute the risk index for each calf. In these sanitary situations, we can see that most calves were exposed to 2 pathogens, while very few were exposed to either 0 or 4 (Figure S4, light blue). In addition, we also generated 10,000 additional sanitary situations with a positive correlation in the presence of pathogens (Figure S4, yellow). In this case, we can see that a substantial amount of calves was exposed to either 0 or 4 pathogens at their cow-calf producer.

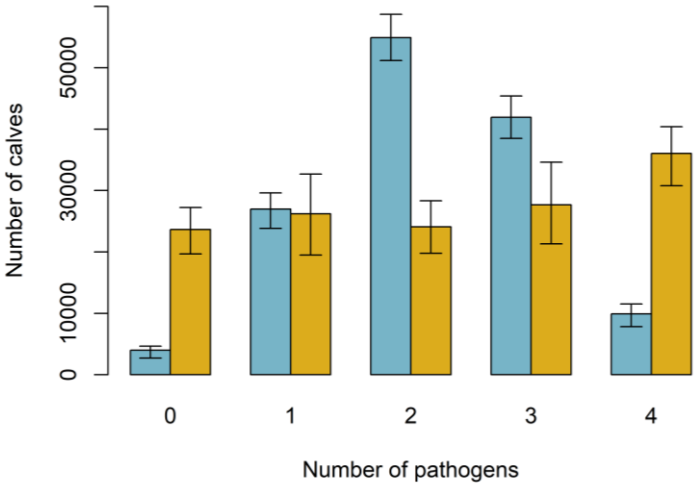

Figure S4: Distribution of the number of pathogens carried by the calves in a case with (yellow) or without correlation (light blue). The bars represent the first and fourth quartile of the distribution of the random draws.

However, this change in the distribution of pathogens among the cow-calf producers (and therefore among the calves) had little impact on the values of $R_{i,\cdot,hist}$ and $R_{i,\cdot,opti}$ (Figure S6). First, there was a very strong correlation between the average risk index of a same calf with or without correlation, whether it was for the batch composition from the database from *Terrena Production Bovine* (Spearman's ρ = 0.985) or for the batch composition derived from the algorithm (Spearman's ρ = 0.992). Besides, the distribution of the risk indices is very close to the distribution obtained without correlation presented in the main text of the article. Notably, the mean risk indices remain almost unchanged ($R_{i,\cdot,hist}$ = 21.55 with correlation and $R_{i,\cdot,hist}$ = 21.53 without ; $R_{i,\cdot,opti}$ = 20.08 with correlation and $R_{i,\cdot,hist}$ = 20.07 without).

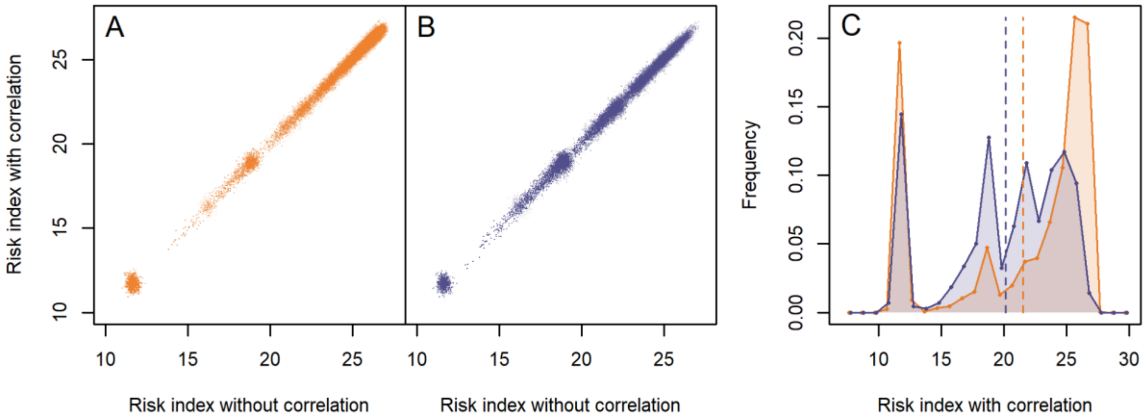

Figure S5: A and B: Risk index with correlation as a function of the risk index without correlation for each calf in the database from *Terrena Production Bovine*, with the batch composition form the database (A, orange) and from the algorithm (B, purple). C: Distribution of the risk indices for the historical (orange) and optimized batch compositions (purple), with correlation.
