# Supplementary material for: Minimizing the number of origins in batches of weaned calves to reduce their risks of developing bovine respiratory diseases
Source: Vet Res. 2021 Jan 7;52:5. doi: 10.1186/s13567-020-00872-z (PMC7792323; doi:10.1186/s13567-020-00872-z)
Supplement: Supplementary file 3 — Additional file 3. Impact of the algorithm on the risk index depending on batch characteristics. [file 13567_2020_872_MOESM3_ESM.pdf]

Additional file 3: Impact of the algorithm on the risk index depending on batch characteristics

In order to better understand how the algorithm impacted the risk index of calves depending on the characteristics of the batches they were initially assigned to, we divided our set of batches into classes according to four different batch characteristics:

- Four classes of batch sizes, each including 25% of the calves: [1;12], [13;31], [22;32] and [31;132].
- Four classes of proportion of calves of the main breed in each batch corresponding to those presented in the main text of the article: [0;0.5[(3% of the batches), [0.5;0.9[(24%), [0.9;1[(11%) and 1 (61%).
- Four classes of number of origins of batches according to the database from *Terrena Production Bovine*, each including 25% of the calves: [1;2], [3;6], [7;10], [11:31].
- Four classes of standard deviation of weight (in kg) of batches according to the database from *Terrena Production Bovine*, each including 25% of the calves: [1;14], [15;21], [22;29] and [30;221].

Then, we computed for each class the average values of $R_{i,\cdot,hist}$ and $R_{i,\cdot,opti}$, i.e. the risk indices averaged over all the sanitary situations and the calves of a same class (Figure S6).

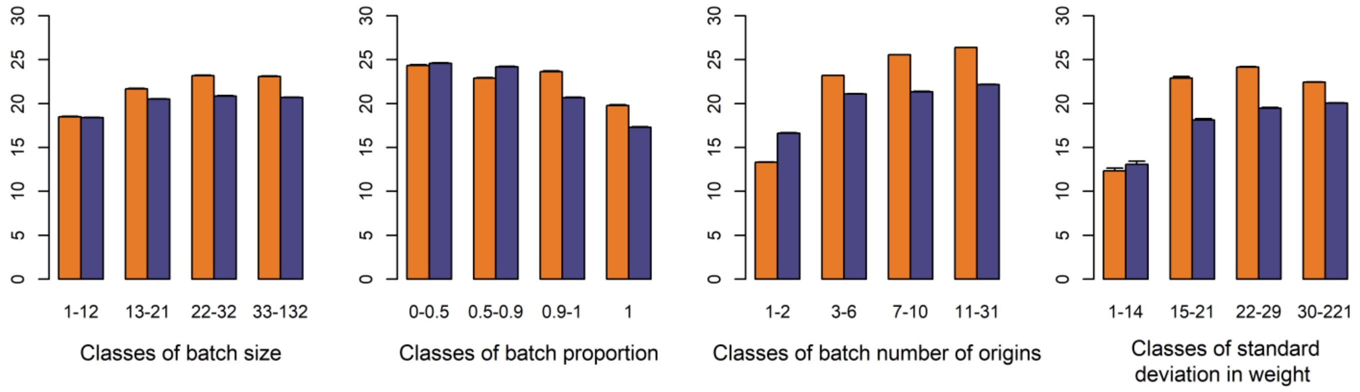

Figure S6: Average values of $R_{i,\cdot,hist}$ (orange) and $R_{i,\cdot,opti}$ (purple) over all the calves belonging to a given class of batch size, proportion of calves of the main breed, original number of origins or original standard deviation in weight (from left to right). Error bars represent 2 times the standard error of the mean.

First, results showed that the algorithm limited the increase in risk index of the calves with the batch size: there was almost no difference for the lowest class (-0.1 for class [1;12]), but a larger difference for the highest class (-2.4 for class [31;132]).

Second, the algorithm slightly increased on average the risk index of the batches with a low proportion of calves of the main breed (+0.2 for class [0;0.5[and +1.3 for class [0.5;0.9[) while it strongly reduced the risk index of the calves in batches with a high proportion of calves of the main breed (-2.9 for class [0.9;1 and -2.5 for class 1).

Third, the algorithm also decreased the risk index for the three largest classes of number of origins according to the database (notably -4.1 for class [11-31]). However, it also increased the risk index of the batches with the lowest number of origins (+3.3 for class [1;2]), although the average of $R_{i,\cdot,opti}$ of all the calves in the batches of the class [1;2] remained lower (17.5) than the average for the class [3;6] (20.6)

Finally, the algorithm also reduced the risk indices in the batches with the largest weight heterogeneity ([15;21], [22;29] and [30;221]), but slightly increased the average risk index for the calves in the most homogeneous batches in weight (+0.7 for class [1;14]).
