# Supplementary material for: Minimizing the number of origins in batches of weaned calves to reduce their risks of developing bovine respiratory diseases
Source: Vet Res. 2021 Jan 7;52:5. doi: 10.1186/s13567-020-00872-z (PMC7792323; doi:10.1186/s13567-020-00872-z)
Supplement: Supplementary file 4 — Additional file 4. Robustness of the results to changes in parameter values. [file 13567_2020_872_MOESM4_ESM.pdf]

Additional file 5: Robustness of the results to changes in parameter values

We computed the risk indices of the calves, for the batch compositions from the database from *Terrena Production Bovine* ($R_{i,\cdot,hist}$) and the one from the algorithm ($R_{i,\cdot,opti}$), with values of the pathogen parameters (α_k, β_k, γ_k and δ_k) either doubled or halved (Figure S7). Since $\delta_k = 0$ for BRSV and PI-3, we did not considered the variations of these parameters.

Results show variations in the absolute values of the risk indices, with generally a translation of the distributions towards higher values when a parameter is doubled and a translation towards lower values when a parameter is halved. However, the differences between the distribution of $R_{i,\cdot,hist}$ and the distribution of $R_{i,\cdot,opti}$ are conserved.

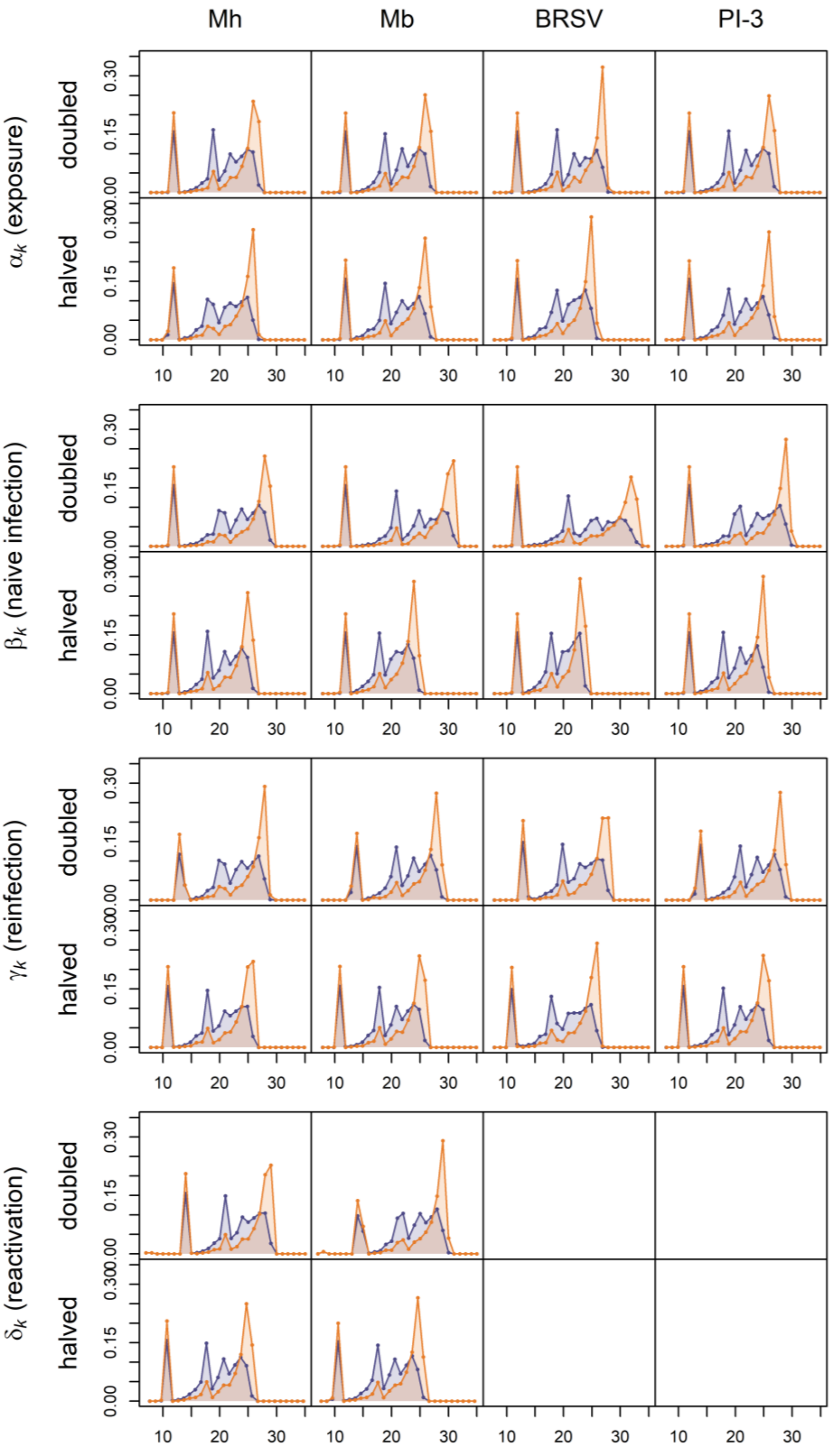

Figure S7: Distributions of $R_{i,\cdot,hist}$ (in orange) and $R_{i,\cdot,opti}$ (in purple) with one of the four pathogen parameters of one of the pathogens either doubled or halved.
